# Assessment of Naturally Sourced Mineral Clays for the 3D Printing of Biopolymer-Based Nanocomposite Inks

**DOI:** 10.3390/nano11030703

**Published:** 2021-03-11

**Authors:** Rebeca Leu Alexa, Horia Iovu, Bogdan Trica, Catalin Zaharia, Andrada Serafim, Elvira Alexandrescu, Ionut-Cristian Radu, George Vlasceanu, Silviu Preda, Claudia Mihaela Ninciuleanu, Raluca Ianchis

**Affiliations:** 1 Advanced Polymer Materials Group, Department of Bioresources and Polymer Science, Politehnica University of Bucharest, 011061 Bucharest, Romania; leurebeca@gmail.com (R.L.A.); zaharia.catalin@gmail.com (C.Z.); andrada.serafim@gmail.com (A.S.); radu.ionucristian@gmail.com (I.-C.R.); 2 Academy of Romanian Scientists, Splaiul Independentei 54, 050094 Bucharest, Romania; 3 National R-D Institute for Chemistry and Petrochemistry ICECHIM—Bucharest, Splaiul Independentei 202, 6th District, P.O. Box 35/174, 060021 Bucharest, Romania; trica.bogdan@gmail.com (B.T.); elviraalexandrescu@yahoo.com (E.A.); claudia.ninciuleanu@yahoo.com (C.M.N.); 4 Faculty of Medical Engineering, University Politehnica of Bucharest, Gheorghe Polizu 1-7, 011061 Bucharest, Romania; vlasceanu.georgemihail@yahoo.ro; 5 Institute of Physical Chemistry “Ilie Murgulescu”, Romanian Academy, Splaiul Independentei 202, 6th District, 060021 Bucharest, Romania; predas01@yahoo.co.uk

**Keywords:** nanocomposites, nanoclay, biopolymer, hydrogel, inks, 3D printing

## Abstract

The present study investigated the possibility of obtaining 3D printed composite constructs using biomaterial-based nanocomposite inks. The biopolymeric matrix consisted of methacrylated gelatin (GelMA). Several types of nanoclay were added as the inorganic component. Our aim was to investigate the influence of clay type on the rheological behavior of ink formulations and to determine the morphological and structural properties of the resulting crosslinked hydrogel-based nanomaterials. Moreover, through the inclusion of nanoclays, our goal was to improve the printability and shape fidelity of nanocomposite scaffolds. The viscosity of all ink formulations was greater in the presence of inorganic nanoparticles as shear thinning occurred with increased shear rate. Hydrogel nanocomposites presented predominantly elastic rather than viscous behavior as the materials were crosslinked which led to improved mechanical properties. The inclusion of nanoclays in the biopolymeric matrix limited hydrogel swelling due the physical barrier effect but also because of the supplementary crosslinks induced by the clay layers. The distribution of inorganic filler within the GelMA-based hydrogels led to higher porosities as a consequence of their interaction with the biopolymeric ink. The present study could be useful for the development of soft nanomaterials foreseen for the additive manufacturing of customized implants for tissue engineering.

## 1. Introduction

As 3D fabrication technologies continue to mature, the development of new and efficient printing inks is to be expected as industrial players continuously look for new products that surpass the limitations of the existing ones. Recent advancements in printing inks for tissue engineering based on biocompatible materials have been focused on developing optimum 3D printed materials with controlled bulk geometry and internal structure [1,2,3,4,5,6]. Several hydrogel-based inks have been investigated for 3D plotting due to their excellent biocompatibility and physiological degradation, printability, high swelling and crosslinking capacity, characteristics which render them as an attractive class of materials for 3D cell culture [2,5,6,7,8]. In order to be used as biomaterial inks, several requirements have to be considered for injectable hydrogel inks, such as: flow under low pressures, rapid gelation kinetics and dimensional stability following extrusion [9]. The production of hydrogels may be accomplished by physical crosslinking, chemical gelling or assembly, on both synthetic and natural polymers. Additionally, combinations thereof may be used in order to obtain end-use-specific properties. Thus, hydrogels based on different natural polymers (alginate [10,11], gelatin [12,13], cellulose [14,15], collagen [16,17,18], fibrinogen [19,20], hyaluronic acid [21,22,23]) or synthetic polymers (such as polyacrylamide [24,25], polyurethane [26,27,28,29], poly-(ethylene glycol) [30,31]) have been explored for the development of biomaterial inks. Naturally sourced bioactive polymers demonstrate superior biofunctionality over synthetic ones. Furthermore, they exhibit low immune system stimulation, bioresorbability and remarkable biocompatibility [4,32,33]. Among them, gelatin, a water-soluble protein derived from collagen, is one of the most used and well-defined biomaterials in tissue engineering applications. Due to its properties, such as high biocompatibility and biodegradability, considerable crosslinking potential and good thermal stability in physiological environments, but also cost efficiency, gelatin is considered to be one of the most popular biopolymers used in the development of 3D printing inks [31,32]. Moreover, gelatin hydrogels exhibit essential morphological properties, which allow cell attachment and spread within gelatin scaffolds. Proliferation and differentiation are essential processes for tissue behavior and its function determination. Due to its special structure, gelatin presents cell-binding moieties like arginine–glycine–aspartic acid (RGD) sequences [33]. Gelatin-based inks are suitable for 3D printing of transplantable scaffolds of tissues, ranging from skin to bone or cartilage [10,13,34,35,36,37].

However, 3D printing with soft materials comes with a range of challenges, mostly because of poor mechanical strength of the printed materials, limiting, therefore, their application. In this respect, hydrogel nanocomposite-based inks are promising materials for tissue engineering applications. They combine the advantages of such components and highlight various requirements, including bioactivity, mechanical strength, controllable and tailorable degradation properties and a facile preparation process [4,5,6,31].

Among the great diversity of nanocomposite materials, polymer–clay systems can be classified as an emerging class due the tremendous application potential. The 2D inorganic nanofiller type of clay can have applications in several domains, such as the automotive industry, environmental protection, electronics, packaging, cosmetics, therapeutic delivery and medicine [7,38,39,40,41].

Recently, 3D printing of scaffolds for tissue engineering was demonstrated to be another promising research pathway where clays have found their relevance. Some researchers suggested that clays, such as montmorillonite and kaolinite, may have been important actors in the processes leading to the appearance of life because of their ability to retain organic molecules within their layered structure, supporting the formation and replication of biopolymers [42]. Clay is considered the raw material for the creation of human life by several religions [43]. Over the years, several studies demonstrated that nanoclay particles possess essential valuable features for regenerative medicine, such as cell adhesion and spreading but also the ability to modulate the drug release [44,45,46].

Clays proved to be excellent fillers for hydrogels designed as inks for 3D fabrication applications, with clay nanolayers inducing an increased viscosity of polymeric ink and stabilizing the polymer network [13,46,47,48]. The addition of synthetic clay improved printability and increased shape fidelity of alginate-based inks due polymer–clay reversible interactions. Moreover, biologically active loaded bioink showed a more sustained release profile from the printed constructs in the presence of nanoclay [47,49].

After a careful search on PubMed and Google Scholar engines using *3D print* or *additive manufacturing* and *clay/layered silicate/laponite/natural clay/montmorillonite/bentonite* (each in turn) as keywords, twenty-six relevant articles on 3D printing using clay-containing inks were found. The vast majority of the studies used synthetic clays (Laponite XLG or XLS) as the inorganic components of the polymeric ink. Among these studies, only six referred to the inclusion of natural clay, namely, commercial organically modified clay, Cloisite 30B, in the printing ink. None of the studies referred to hydrogel nanocomposite inks, but to epoxy, polylactic acid and acrilonitril–butadiene–styrene-based polymeric inks.

Indeed, Laponite could be a very good choice when compared to natural clays because synthetic clays display advanced purity, but also controllable crystal dimensions and uniform dispersion. However, natural clays could also be suitable candidates to be used in additive manufacturing. One of the most important properties that makes them suitable is that they are naturally available resources with high availability. Therefore, time, substances and energy consumption could be reduced when fabricating printing ink using natural clay.

Montmorillonite natural clay (MMT) is a major component of bentonite, an already Food and Drug Administration (FDA)-approved additive with usage in medicinal products [45]. MMT belongs to the smectite family, being a plate-shaped layered nanosilicate of ~1 nm in thickness and 0.2–2 μm in diameter. The MMT structure consists in one alumina octahedral sheet placed between two silicon tetrahedral layers with the general formula [(Na,Ca)_0_._33_(Al,Mg)_2_Si_4_O_10_(OH)_2_·nH_2_O]. MMT nanoparticles were demonstrated to adsorb bioactive molecules due to their large and highly charged plane surface area [50]. The intercalation of hydrophobic polymers into inter-layer spaces of hydrophilic clays is difficult to achieve [51]. In this respect, nanoclays can be functionalized with hydrophobic compounds. By organomodification, clay nanoplatelets became amphiphilic and thus compatible with the polymer matrices also acting as delivery vehicles for several organic molecules [50,52,53,54,55,56]. Additionally, clays are osteoinductive silica-rich inorganic species and, when investigated for skeletal regeneration, were shown to promote osteogenic differentiation and increased network stiffness and porosity [46,47,48]. Their unique features make natural clays promising candidates in tissue engineering. The incorporation of functionalized natural clay nanoparticles into hydrogel inks/3D printed scaffolds could lead to a sustained release of different polar/non-polar drugs at the target sites [44,45,52,54,55,56,57].

The present study investigated the possibility of developing 3D printed composite constructs using a biomaterial-based nanocomposite ink containing several types of nanoclay. Methacrylated gelatin (GelMA) was used as the biopolymeric matrix and three types of clays as reinforcing fillers. Our aim was to decrease the polymeric content of the printing ink and future 3D construct, while preserving printing fidelity. The physico-chemical behavior of methacrylated gelatin in the presence of several types of hydrophobically modified clay was followed by and compared with unmodified natural clay loaded with GelMA ink/material but also with the neat GelMA ink. To our knowledge, this is the first systematic study which investigates natural clay and functionalized natural clay inclusion in GelMA-based printing inks. In order to estimate their suitability for new personalized bioactive medical devices, a comparative evaluation of these new types of nanocomposite inks was performed. These findings would allow the simultaneous release of hydrophilic/hydrophobic bioactive agents from the customized 3D scaffold, in a controlled manner. To the best of our knowledge, this study represents a premiere for the community of 3D printing of soft materials.

## 2. Materials and Methods

### 2.1. Materials

Methacrylic anhydride (MA) (MW = 154.16 g/mol) (Sigma-Aldrich, Goettingen, Germany), 2-Hydroxy-4′-(2hydroxyethoxy)-2-methylpropiophenone (Irgacure-2959) (MW = 224.25 g/mol) (Sigma-Aldrich, Milano, Italy) and gelatin (from bovine skin, gel strength ~225 g Bloom, type B) was purchased from Sigma-Aldrich St. Louis, Missouri, USA, and were used as received. Commercial clays (Cloisite Na, Cloisite 30B and Cloisite 15A) were kindly offered by Southern Clay Products Inc. (Gonzales, Gonzales, TX, USA) and were used as received. Cloisite Na corresponds to the commercial name of natural montmorillonite. Cloisite 30B and Cloisite 15A represent the commercial names for natural montmorillonite modified with quaternary ammonium salts: methyl, tallow, bis-2-hidroxyethyl, quaternary ammonium—Cloisite 30B and dimethyl, dihydrogenated tallow, quaternary amoonium—Cloisite 15A.

Phosphate-buffered solution (PBS) pH = 7.4 was prepared in our laboratory. Ultrapure water was used as a dispersion medium.

### 2.2. Experimental

GelMA with an 80% degree of metacrylation was synthesized according to well-established protocols. Briefly, 10 g of gelatin were solubilized in 90 mL PBS (pH = 7.4) at 50 °C in order to obtain a homogenous solution. Then, 3 mL of methacrylic anhydride were added drop by drop into the gelatin solution. The pH was controlled by adding a certain amount of sodium hydroxide (NaOH) solution. In order to allow for the methacrylic anhydride to react with functional groups in the protein structure, the reaction was maintained at 50 °C for 2 h under vigorous magnetic stirring. After the reaction was complete, the solution was dialyzed against distilled water in dialysis cellulose bags (MWCO = 8000 Da) for 8 days at 40 °C in order to remove the unreacted MA and the secondary products formed during the reaction process. Finally, functionalized gelatin was dried by lyophilization at 0.28 bar for 48 h (D-37520, Osterode am Harz, Germany) and stored at room temperature.

### 2.3. Preparation of the Hydrogel

GelMA neat printing ink was prepared by dissolving lyophilized GelMA at a concentration of 20% (*w*/*v*) with a degree of methacrylation of 80%, in PBS (pH = 7.4) at 40 °C, under gentle stirring. Following GelMA dissolution, the photoinitiator was added. Furthermore, the solution was homogenized under magnetic stirring at 40 °C.

In order to achieve the nanocomposite hydrogel-based printing inks, we selected a fixed concentration of natural clay (3% *w*/*w* to GelMA). Then, three biomaterial-based printing inks/nanomaterials were prepared by modifying GelMA with commercial montmorillonite clays, namely, natural clay (Cloisite Na) and organomodified clays (Closite 30B and Closite 15A).

Firstly, clay powder (Cl Na, Cl 30B or Cl 15A, respectively) was dispersed under magnetic stirring in PBS (pH = 7.4). After 12 h, lyophilized GelMA (20% *w*/*v*) was added to the dispersion and allowed to dissolve under gentle stirring at 40 °C. After the mixture became homogenous, the photoinitiator (Irgacure-2959, 1% (*w*/*w*) to GelMA) was added. The resulting viscous solution was subjected to magnetic stirring at 40 °C until homogenization.

### 2.4. ^1^H-NMR Spectrometry of Gelatin and Gelatin Methacrylate (GelMA)

In order to determine the methacrylation degree of GelMA, ^1^H-NMR spectrometry was used. The spectrum was obtained using a Bruker NMR 600 MHz Advance instrument, and the methacrylation degree (MD) was calculated using Equation (1) [58]:

(1) $$MD\left(\%\right)=1-\frac{\mathrm{integration}\;\mathrm{signal}\;\mathrm{of}\;\mathrm{arginine}\;\mathrm{from}\;\mathrm{GelMA}}{\mathrm{integration}\;\mathrm{signal}\;\mathrm{of}\;\mathrm{arginine}\;\mathrm{from}\;\mathrm{GelMA}}\times 100$$

In order to determine the number of amino moles, a dilution was performed and the absorbance was measured. Based on the calibration curve, the number of experimental moles was determined from Equation (2).

(2) $$DS\left(\%\right)=1-\frac{y\times 100}{\frac{X\times 100}{mp}}\times 100$$

where *X* = number of amino moles of GelMA and *y* = number of amino moles of gelatin.

### 2.5. 3D Printing of GelMA-Based Inks

A 3D Discovery™ printing machine (RegenHU Ltd., Villaz-St-Pierre, Switzerland) was used to print the GelMA-based inks. The additive manufacturing process was performed using a direct dispensing print head.

Using BioCAD, square scaffolds were drawn. Scaffolds were printed at room temperature. In order to establish the suitable printing parameters, different printing speeds, ranging from 3 mm/s to 11 mm/s, and pressures in the range of 150–300 kPa, were explored. The scaffolds were UV cured at 360 nm.

### 2.6. Fourier Transform Infrared Spectrometry with Attenuated Total Reflectance Accessory (ATR-FTIR)

The samples were analyzed as powders using a Vertex 70 Bruker FTIR spectrometer. For all the formulations, the FTIR spectra were registered at a resolution of 2 cm^−1^ in the 4000–400 cm^−1^ wavenumber region.

### 2.7. Scanning Electron Microscopy (SEM) and Micro-Computed Tomography (Micro-CT)

Morphological information, including internal structure, was obtained by environmental scanning electron microscopy (ESEM-FEI Quanta 200, Eindhoven, The Netherlands) of the lyophilized samples.

Micro-CT analysis was performed on lyophilized samples using a Bruker microCT 1172 high-resolution micro-CT scanner. For micro-CT analysis, one intersection of the printed filaments from each composition was analyzed. The scanned datasets were aquired at a resolution of 5.4 µm, with no filter, at a source voltage of 60 kV and a current intensity set at 150 µA, during 180° rotations of the sample, with a rotation step of 0.3° and frame exposure of 1150 ms, as the average of 6 successive acquisitions. Bruker NRecon, Bruker CTVox and Bruker CTAn software was used to reconstruct, depict and determine the quantitative porosity features. For each of the three composite prints, one rectangular volume of interest (VOI) dataset was extracted, constrained in terms of size. In CTAn, the VOIs were processed through a routine consisting of thresholding, despeckling and 3D analysis to quantify pore distribution.

### 2.8. X-ray Diffraction (XRD) and Transmission Electron Microscopy (TEM)

The structure of the samples was determined using an X-ray diffractometer (Rigaku Ultima IV, Tokyo, Japan) with CuKα radiation (λ = 1.5406 Å), operated at 40 kV and 30 mA. The analysis was run in continuous mode, at room temperature and atmospheric pressure. The data were collected over the 2θ range 1–50° and a scanning speed of 1°/min. The samples were measured in a powder state.

TEM analysis was accomplished on the lyophilized nanocomposite scaffold specimens. Samples were analyzed in BF-TEM (Bright Field Transmission Electron Microscopy) mode at an accelerating voltage of 200 kV on a TECNAI F20 G² TWIN Cryo-TEM (FEI, Hillsboro, Oregon, USA). For each sample, the distance between non-intersecting layers was measured in random positions on the grid. For each sample, a representative group of values for the interlamellar spacing was obtained. The Shapiro–Wilk normality test was applied on all batches of data. For reasons of non-normality, the differences between the groups were evaluated statistically in R software by the Kruskal–Wallis rank sum test. Dunn’s test for multiple comparisons was used to evaluate the pairwise differences between the groups.

### 2.9. Rheological and Mechanical Analyses 

The rheological behavior of the synthesized ink formulations was investigated using a Kinexus Pro Rheometer (Malvern, Worcestershire, UK) equipped with cone–plate geometry (angle of 4° and a diameter of 40 mm) and a Peltier element for precise control of the temperature. Samples dehydration was prevented by using a water lock. The tests were performed at a shear rate interval of 0.1 ÷ 1000 s^−1^ at a constant temperature of 37 °C. All the samples were examined in triplicate and the average values are represented in the graphics.

Mechanical properties of the crosslinked 3D printed hydrogels were studied using the nanoindentation technique. Nanoindentation analyses were performed on a Nano Indenter^®^ G200 (Santa Clara, CA, USA), and a G-Series DCM CSM Flat Punch Complex Modulus Gel was used. This method allows for determining the complex shear modulus G*, storage modulus G’ and loss modulus G’ of gels very quickly at a single frequency. The principle of the indentation test is as follows. First, the test is initiated by the system moving the sample from the microscope to the DCM II head. Afterwards, for each indentation, the sample is moved automatically under the indenter tip. Finally, when all the analyses are complete, the sample is moved by the system under the microscope.

### 2.10. Wettability, Swelling Degree, Degradability of the 3D Printed Hydrogel Based on GelMA

Water contact angle measurements were performed on pellet samples in triplicate and were determined using a DSA100 drop shape analyzer equipped with a charged-coupled (CCD) video camera. The water contact angles were automatically calculated by the software of the instrument analyzer.

For swelling degree studies, the 3D printed lyophilized samples were measured as follows. Each sample was immersed in PBS (pH = 7.4) at 37 °C and weighed after predetermined periods of time. The swelling degree (SD) was calculated using the following equation:

(3) $$SD\left(\%\right)=\frac{\;\left(Ww-Wd\right)}{Wd}\times 100$$

where *W_w_* = wet weight, *W_d_* = dry weight.

Dissolvability measurements were performed on the 3D printed lyophilized samples immersed in PBS (pH = 7.4) at 37 °C. After incubation, the samples were dried by lyophilization. The resulting samples were weighed and the relative weight (DR) was defined as follows:

(4) $$Dissolvability\left(\%\right)=\frac{W_{0}-Wd}{W_{0}}\times 100\;$$

where *W_d_* represents the weight of the remaining dried samples after 72 h and *W*_0_ the weight of dried hydrogel samples in the initial state. The experiments were performed in triplicate, and all data were averaged over all replicates.

### 2.11. Statistical Analyses

The data are expressed as mean ± SD. The significance of differences was evaluated by one-way ANOVA. Significance was considered at a *p*-value of 0.05.

## 3. Results and Discussion

### 3.1. ^1^H-NMR 

The first stage of the present research study was to synthesize methacrylated gelatin (GelMA) through covalent binding of the methacrylate groups on the gelatin surface. The modification of arginine residue units from the gelatin structure with methacrylate groups was confirmed by ^1^H-NMR analysis.

**Figure 1:**
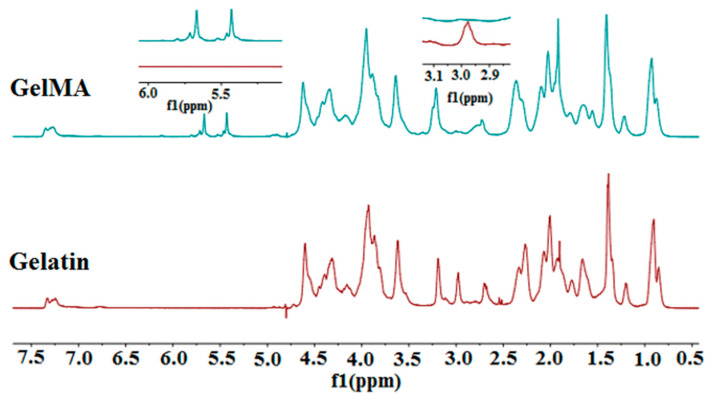
^1^H-NMR spectra of methacrylated gelatin (GelMA) and neat gelatin.

^1^H-NMR spectra of GelMA (Figure 1) revealed two new signals at δ = 5.4 ppm and δ = 5.6 ppm that correspond to the acrylic protons of methacrylic functions. These methacrylic functions are characteristic of the MA structure, which was grafted onto the gelatin backbone during the reaction between gelatin–arginine residues and methacrylic anhydride [59]. The methacrylation was also confirmed by the appearance of the methyl group signal at 1.8 ppm and by the decrease in the amino group signal at 2.9–3 ppm [60]. The degree of methacrylation was calculated using amine signals and, as an internal reference (used in order to normalize the amine signals (2.9 ppm) of methacrylated aminoacids), phenylalanine signals were set (7.0–7.5 ppm) for five protons [61]. The methacrylation degree of GelMA was determined to be 85%.

^1^H-NMR results were confirmed by UV analysis. The amino group dosing procedure is based on an initial calibration between ninhydrin and an amino compound (glycine). Based on the literature, an average molecular mass of the structural units in the gelatin chain was calculated [58]. Based on the determined average molecular weight and the percentages of amino groups in lysine and arginine, the number of theoretical moles of amino per sample mass was estimated. The number of theoretical moles (0.042 amino moles at 100 g gelatin) allows us to compare the number of moles determined experimentally (0.0236 amino moles at 100 g gelatin).

### 3.2. ATR-FTIR Analyses

**Figure 2:**
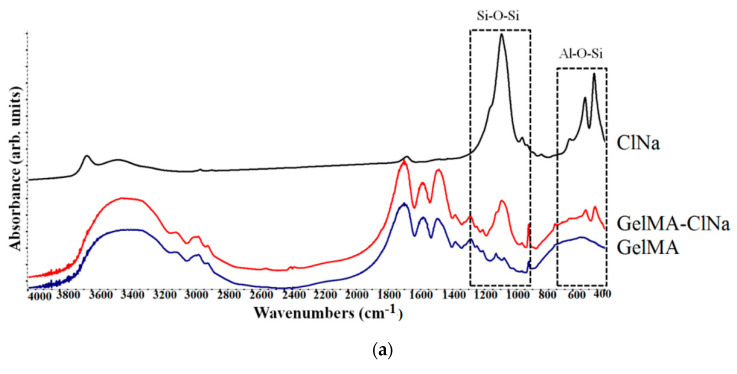

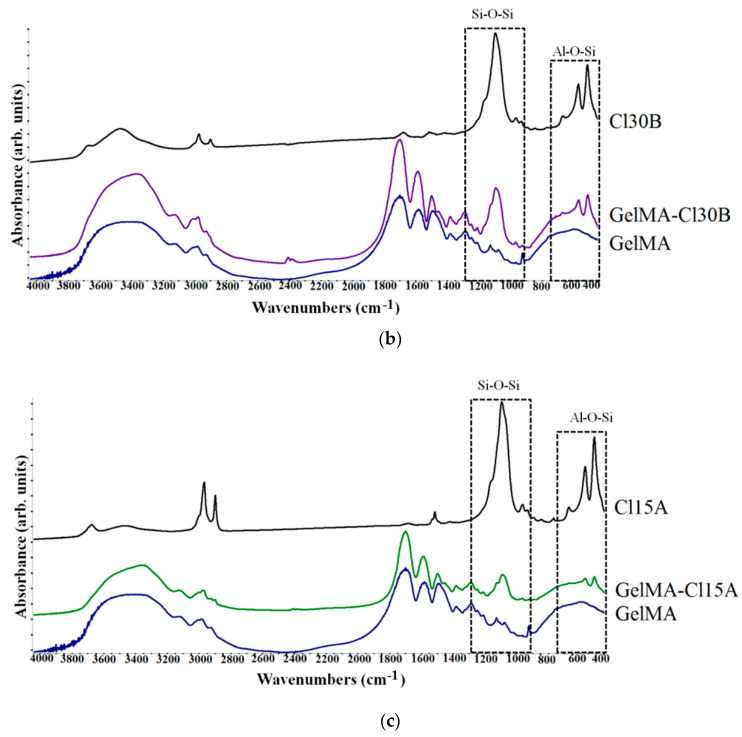
(**a**) FTIR spectra of commercial Cloisite Na, synthesized GelMA–Cloisite Na and GelMA matrix; (**b**) FTIR spectra of commercial Cloisite 30B, synthesized GelMA–Cloisite 30B and GelMA matrix; (**c**) FTIR spectra of commercial Cloisite 15A, synthesized GelMA–Cloisite 15A and GelMA matrix.

The chemical composition and the possible chemical bonds in the structure of nanomaterials obtained in the presence of clay can be verified using FTIR analyses. Therefore, the identification of the functional group characteristics of the clay structure included in the polymeric GelMA matrix was carried out (Figure 2).

FTIR spectra of GelMA-based samples displayed the characteristic absorption bands of functional groups from GelMA and modified GelMA. The absorption bands of functional groups in the GelMA structure are also present in the FTIR spectra of all GelMA nanocomposite samples, being positioned at the same wavenumber. FTIR spectra of GelMA and GelMA modified with clay indicate peaks at 3290–1240 cm^−1^. Peaks observed at 1240 cm^−1^, 1541 cm^−1^ and 1640 cm^−1^ are related to C-N stretching and N-H bending (amide III), to N-H bending (amide II) and to C=O stretching (amide I). These peaks revealed the interaction between gelatin and methacrylate anhydride [62]. The peak at 2934 cm^−1^ is related to CH_2_ groups of alkyl chains. Peaks at 3069 cm^−1^ and 3200–3400 cm^−1^ are related to C-H bonds from amide B and to N-H stretching from amide A. Another peak is observed at 3290 cm^−1^ and is attributed to the stretching of the hydrogen-bonded H-O groups [63,64].

FTIR spectroscopy confirmed the inclusion of clay in the GelMA polymeric matrix. Specific peaks of clay were found in the nanocomposite samples at 1040–1048 cm^−1^ and correspond to Si-O-Si stretching vibration. The next peaks at ~460/520 cm^−1^ are attributed to Al-Si-O vibration [52,65]. FTIR spectra of the nanocomposite samples containing organomodified clays revealed the specific peaks attributed to asymmetric and symmetric stretching vibration of methylene groups at ~2857 cm^−1^. These groups are from the organomodifier agent, namely, hydrocarbon chains of the quaternary ammonium salts [66,67].

### 3.3. XRD and TEM Analyses 

XRD and TEM are the most helpful analyses to find out information about the dispersion of the clay minerals in a polymeric matrix. These techniques allow the identification of the changes in the interbasal distance of the clay, changes that occurred after clay dispersion. The results could indicate the obtaining of intercalated or exfoliated composites with consequences for their final characteristics. Therefore, the dispersion state of ClNa, Cl30B and Cl15A in the GelMA matrix was investigated.

X-ray diffractograms of GelMA samples displayed an amorphous peak at 2θ ~20° [68]. This peak could be associated with the short-range order of protein chains and is present in the XRD profiles of all composite samples.

**Figure 3:**
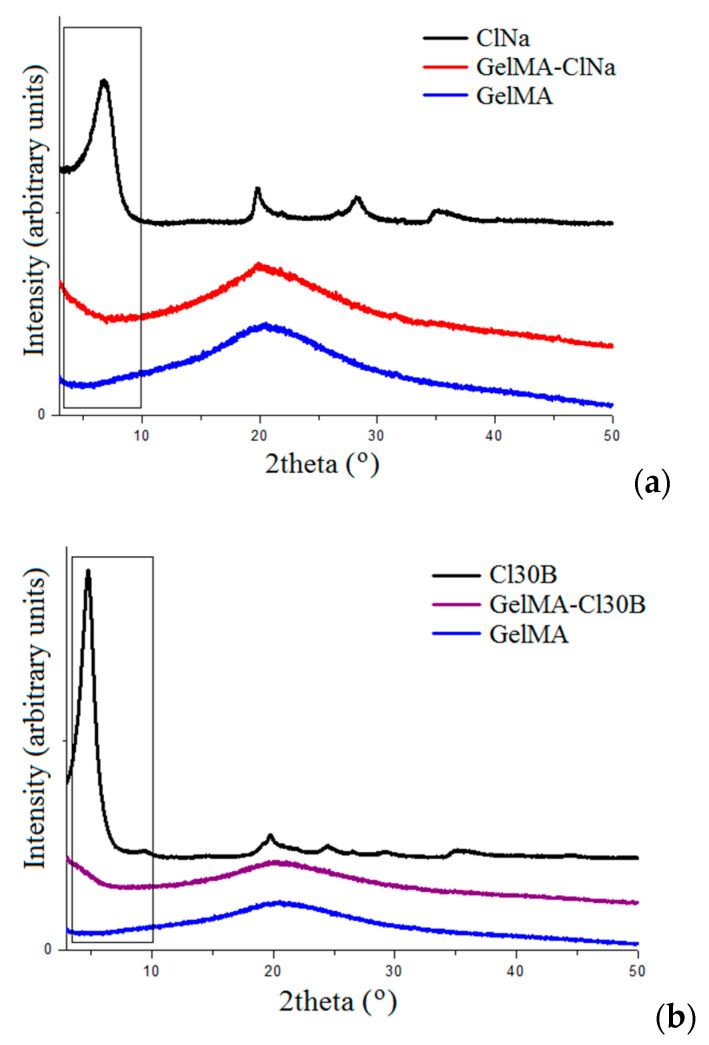

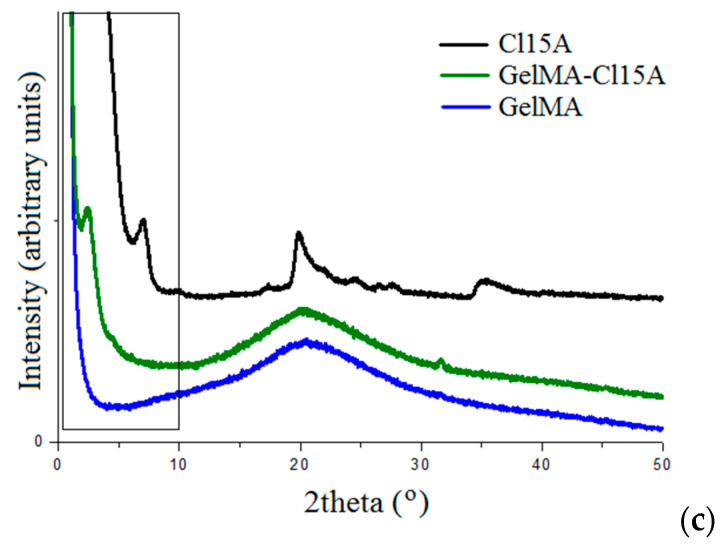
(**a**) X-ray diffractograms of commercial Cloisite Na, synthesized GelMA–Cloisite Na and GelMA matrix; (**b**) X-ray diffractograms of commercial Cloisite 30B, synthesized GelMA–Cloisite 30B and GelMA matrix; (**c**) X-ray diffractograms of commercial Cloisite 15A, synthesized GelMA–Cloisite 15A and GelMA matrix.

The XRD spectra of naturally sourced Cloisites used in the present study showed their characteristic diffraction peaks at 2θ = 7.3° for ClNa, 2θ = 4.9° for Cl 30B and 2θ = 2.7° Cl 15A, corresponding to the interlayer spacing of the crystalline structure d_001_. The change of the clay basal spacing (d_001_) indicates the structure of GelMA–clay nanocomposites. Therefore, intercalation and/or exfoliation of composites was achieved as a consequence of GelMA insertion between silicate nanolayers [44,66]. The XRD profiles of all resulting nanocomposite samples indicated a decrease in 2θ values and, consequently, an increase in the d-spacing. This fact revealed that clays were intercalated/exfoliated into the GelMA matrix. The decrease in intensity and broadening of the ClNa peak in the GelMA-ClNa hydrogels suggests the presence of a mainly exfoliated structure. The Cloisite 30B peak decreased in intensity and sharpness, leading to mostly intercalated composites. As for Cl15A, the characteristic diffraction peaks have a tendency of shifting towards lower angles in the GelMA-Cl15A sample, also indicating an intercalated layered structure (Figure 3).

**Figure 4:**
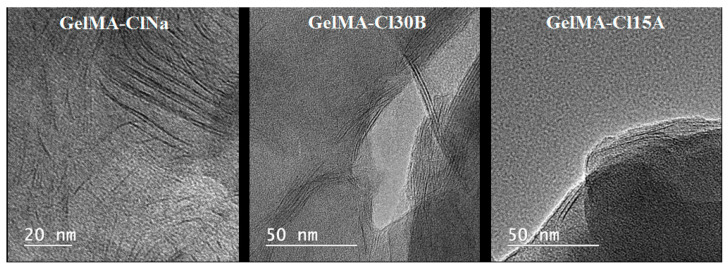
TEM images of synthesized GelMA-based nanocomposites.

TEM images revealed the presence of several areas of dark lines attributed to clay nanosheets distributed almost homogenously in the GelMA matrix (Figure 4). The distancing of the silicate sheets is the result of the penetration of the polymer matrix inside the clay structures; this phenomenon is more evident in Closite Na nanocomposite samples. TEM analyses suggested a generally exfoliated morphology for the prepared Cloisite Na nanocomposite hydrogels, whereas Cloisite 30B and Cloisite 15A formed mainly intercalated structures.

**Figure 5:**
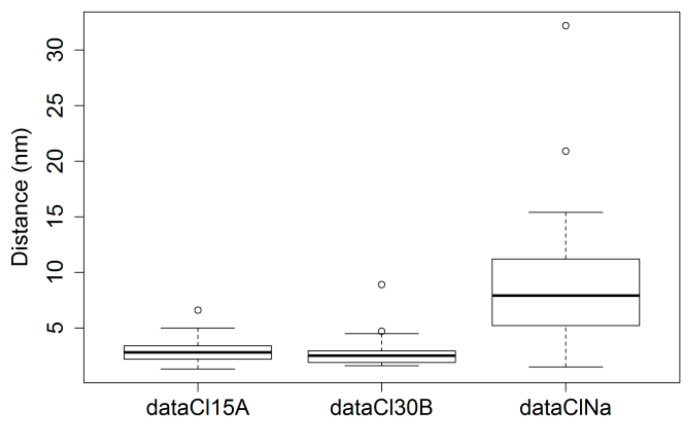
Boxplot representation for the measured distances between neighboring nanosheets for GelMA nanocomposite samples obtained with Cl15A, Cl30B and ClNa; outliers are represented with the circle symbol.

The Shapiro–Wilk test yielded a non-normal distribution of distances measured for GelMA-ClNa (*p*-value = 0.0006) and GelMA-Cl30B (*p*-value = 0.008), for which the test null hypothesis was rejected, and GelMA-Cl15A (*p*-value = 0.23), for which the null hypothesis cannot be rejected. For this reason, the Kruskal–Wallis rank sum test was used to compare the three groups (chi-squared = 45.029, df = 3, *p*-value = 9.1 × 10^−10^). Dunn’s test for multiple comparisons revealed the pairwise differences between the three samples. The boxplots for the three samples are also represented in Figure 5.

As described by the results presented for Dunn’s test for multiple comparisons in Table 1, samples with Cl15A and Cl30B are not statistically different (adjusted *p*-value = 0.283). The null hypothesis of the test cannot be rejected. However, both Cl15A- and Cl30B-based samples are statistically different from the ClNa sample, which is in agreement with XRD results and with the values presented in Table 2 and Table 3 which summarize the datasets. However, when the XRD and the statistical values were compared, some differences could be observed. A possible explanation is that in XRD, the profiles are seen as wide peaks, which suggest an overall distancing of the nanosheets, while in BF-TEM, a more localized distribution is observed. Contrast is also influenced greatly by the orientation of the sheets, which cannot be controlled to a great extent in the BF-TEM analysis.

### 3.4. Rheological and Mechanical Analysis 

**Figure 6:**
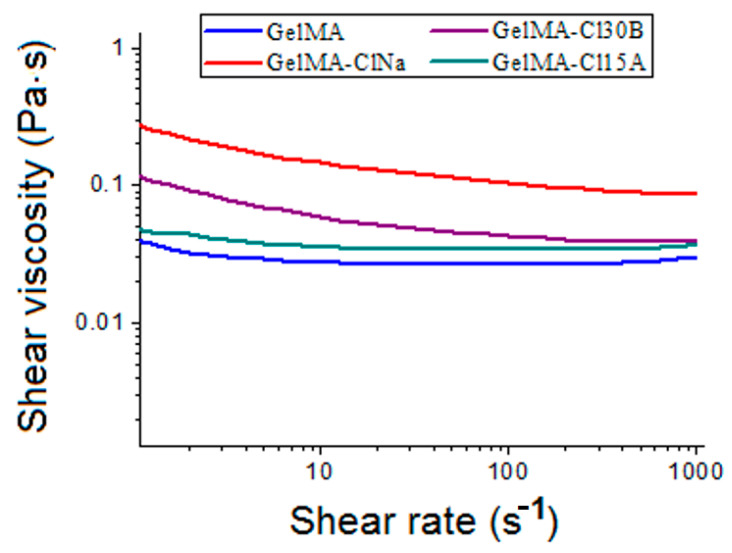
Shear viscosity versus shear rate of inks at 37 °C.

Several studies demonstrated that an intercalated or exfoliated state of clay generates different rheological behaviors of nanocomposite formulations. Conversely, the dispersion state of clay layers in polymers can be evaluated by analyzing the viscosity and the shear thinning exponent [44]. All synthesized inks exhibited shear thinning behavior with increasing shear rate (Figure 6). Increasing shear rate caused the silicate layers to rearrange and align in the flow direction. This phenomenon affected GelMA–clay particle interaction by increasing the free space between dispersed components, which caused the shear thinning behavior detected for all GelMA nanocomposites. At high shear rates (100–1000 s^−1^), the addition in the GelMA solution of Cloisite 15A and Cloisite 30B has a low impact on the viscosity of the precursors, increasing from 0.03 Pa·s for GelMA to 0.037 Pa·s and 0.039 Pa·s for GelMA-Cl15A and GelMA-Cl30B, respectively. However, at low shear rates (0.1 to 100 s^−1^), the addition of Cloisite 15A led to a visible modification of the viscosity of GelMA-Cl15A inks in the shear rate (4.61 Pa·s, while the value registered for GelMA is 2.7 Pa·s). The differences between GelMA-organomodifed clay ink formulations occurred due the different organomodifier concentrations, namely, 90 meq/100 g for Cl30B and 125 meq/100 g for Cl15A [69].

The viscosity of all ink compositions increased with the addition of inorganic particles.

A slight increase in the viscosity of the GelMA-based samples was determined for Cl15A and Cl 30B nanocomposite samples as a result of the intercalation state of clay layers where clay tactoids of larger sizes are formed. Instead, when clay layers exfoliate inside the polymeric matrix, a gel-like structure is generated as a consequence of the large surface area of individual clay layers, leading to an increased viscosity [70,71]. A significant modification of the viscosity was registered for the GelMA-ClNa sample, suggesting that the addition of unmodified clay strongly influences the rheological properties of the mixture in the whole share rate interval.

Therefore, the size and concentration of the dispersed phase, together with the interactions between several type of clays and the GelMA matrix, determined the rheological behavior of the nanocomposite samples. These results were in good agreement with XRD and TEM analyses which revealed the presence of intercalated and exfoliated states of clay layers in the final nanocomposite materials.

**Figure 7:**
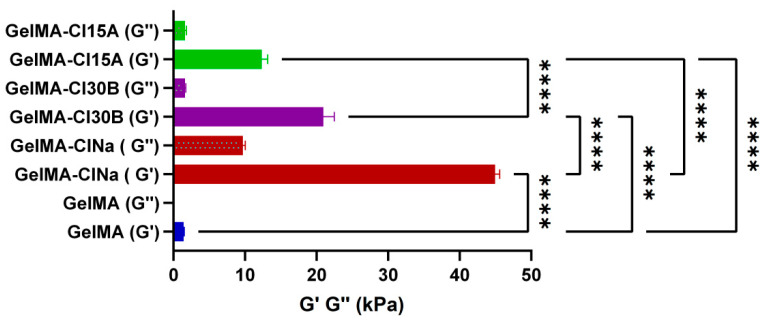
Storage and loss moduli determined by nanoindentation on equilibrium swollen samples. Statistical significance: **** *p* < 0.0001.

The mechanical behavior of the hydrogels obtained after photopolymerization was also examined (Figure 7). The method used to evaluate the hydrogel materials was “G-Series DCM CSM Flat Punch Complex Modulus, Gel” and a flat-ended cylindrical indenter for a DCM II head. A punch with a diameter of 100 µm and Poisson ratio of 0.5 was used.

Generally, studies demonstrated that layered silicates improved the mechanical properties of the final nanomaterials, which were strongly dependent on clay concentration and platelet distribution within the polymeric matrix. In our case, the elastic modulus Gʹ showed higher values for the nanocomposite sample containing ClNa and modified nanoclay when compared to pristine GelMA (Figure 7). GelMA exhibited a value of 1.43 kPa of storage modulus G’, whereas GelMA-ClNa exhibited 44.96KPa, GelMA-Cl30B exhibited 62.85 kPa and GelMA-Cl15A exhibited 37 kPa. This fact indicates an increased mechanical stability and a stronger gel structure, with nanoclay platelets acting as an elastic solid under stress conditions. The results are in good agreement with XRD and TEM morphological analyses where the samples containing ClNa showed mostly exfoliated structures when compared to the other types of nanocomposite samples, GelMA-Cl30B and GelMA-Cl15A. This behavior was probably due to the presence of the hydrophobic modifier, which induced preferential distribution of clay platelets inside the GelMA matrix, causing stack layers or mostly intercalated clay structures, as the morphological studies demonstrated. Additionally, all these samples behaved as crosslinked hydrogels with a storage modulus higher than the loss modulus (G’ > G’’), and had a predominantly elastic rather than viscous character, suggesting complete crosslinking, as can be seen in Figure 7. All these results are in good agreement with other studies conducted on hydrogels and GelMA hydrogels [62,72,73,74].

**Figure 8:**
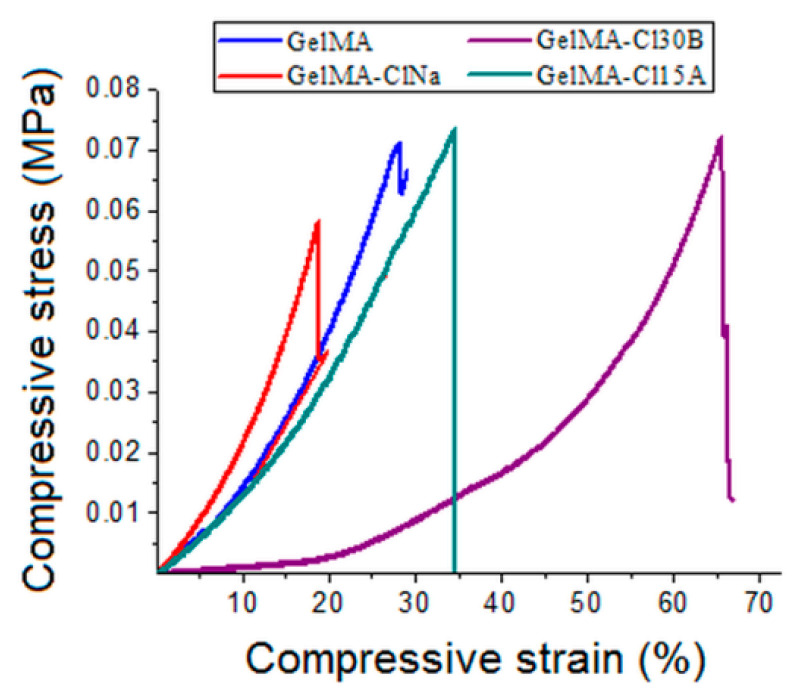
Compressive stress versus compressive strain of samples at equilibrium (37 °C).

The compression tests revealed that the presence of nanofiller led to significant improvements in the mechanical strength of the nanocomposite samples (Figure 8). The presence of nanoclays led to a significant increase in compressive strengths for all of the nanocomposite samples compared to the neat GelMA sample. Under stress conditions, layered silicates tend to increase the intermolecular forces and dissipate the energy in the whole nanomaterial. A higher dispersion of clay nanoplatelets and their interaction with GelMA had direct consequences on the crosslinking process and on the compressive strength of the final hydrogel-based nanocomposite materials. The nanocomposite samples with Cl15A and Cl30B displayed the highest increase in the compression strength (73 kPa for GelMA-Cl15A and 72 kPa for GelMA-Cl30B) compared to the other samples (65 kPa for GelMA and 57 kPa for GelMA-ClNa). Organomodified nanoclays seem to generate stronger nanocomposite systems, probably because of an additional compatibility amongst hydrophobic groups of modified clay and methacrylic groups from GelMA networks. The organic groups may also induce a slight elasticity which may recover better from the applied mechanical stress.

### 3.5. Contact Angle Measurements, Swelling and Degradation Studies

**Figure 9:**
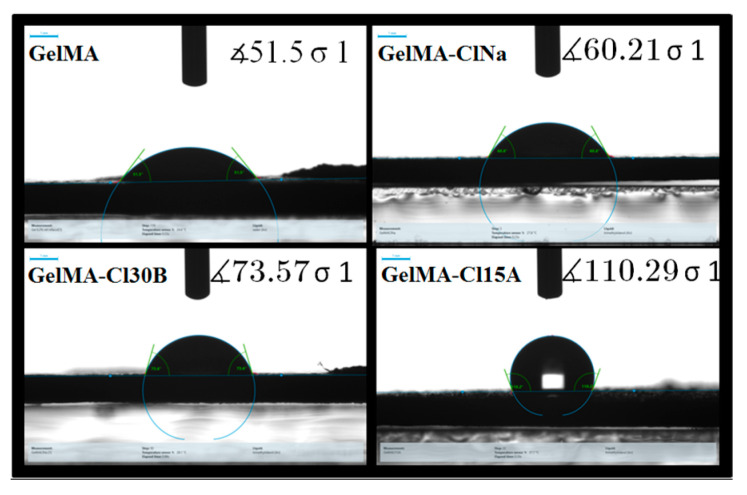
Water contact angle measurements for GelMA and GelMA-based samples.

Hydrophilic/hydrophobic character is another important feature to be studied because it highlights the surface properties of the hydrogel materials which are intended for tissue engineering applications. Therefore, contact angle and swelling degree analyses were carried out on the nanocomposite sample against a neat GelMA sample (Figure 9). Meanwhile, our study focused on the influence of clay type on these parameters, as former studies demonstrated that the methacrylation degree, concentration of GelMA and the amount of nanoclay or photoinitiator added in GelMA solutions played an important role and determined the behavior of the final materials.

**Figure 10:**
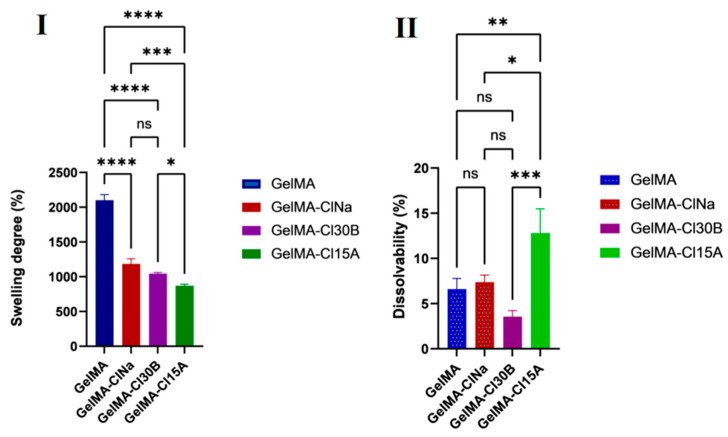
Swelling (**I**) and dissolvability (**II**) analyses of GelMA, GelMA-ClNa, GelMA-Cl30B, GelMA-Cl15A 3D printed scaffolds. (**I**) Statistical significance: ns *p* < 0.5; * *p* < 0.05; *** *p* < 0.0005; **** *p* < 0.0001; (**II**) statistical significance: ns *p* < 0.5; * *p* < 0.05; ** *p* < 0.005; *** *p* < 0.0005.

All nanocomposite samples presented a variation in hydrophilicity when compared to the biopolymeric matrix. With the introduction of the nanoclays in the GelMA matrix, the contact angle of the samples increased. This effect was attributed, firstly, to the chemical composition and, secondly, to the physical barrier effect induced by the clay layers. The “tortuous path” created by the clay layers limited the diffusion of water molecules through the nanocomposite samples, demonstrating an increase in water contact angles values against the GelMA sample [75,76]. When organophilic clays were included, the water contact angles were higher than those obtained for pure GelMA and GelMA-ClNa samples, most likely due to the presence of clay organomodifiers which reduced the sensitivity to water of the nanocomposite tablets. These organomodifiers are fatty acids which usually serve as hydrophobic compatibility agents [77]. These assumptions correlated very well with the swelling studies where decreased values of the swelling degree were obtained for the GelMA-based sample obtained in the presence of organomodified clays (Figure 10I). From another point of view, the inclusion of clay in the GelMA matrix and therefore the replacement of the polymer with clay did not have the same effect as GelMA on the degree of swelling. Likewise, this behavior can be explained by the physical barrier effect induced by the clusters formed by clay layers, leading to the limitation of hydrogel swelling.

The dissolvability tests of GelMA samples were influenced by the presence of the clay nanoparticles (Figure 10II) which, generally, emphasized the degradation of nanocomposite hydrogels. Degradation is typically mediated by hydrolysis and can occur in the polymer backbone or at the crosslinks. Thus, the detachment of the clay–hydrogel microaggregates occured with a greater speed than GelMA network degradation. Surprisingly, the lowest dissolvability was calculated for the sample obtained in the presence of Cl30B. It was reported earlier that this type of clay sometimes interacts differently with polymer matrices than with other types of clay. This behavior was attributed to the hydroxyl groups from the organic modifier of Cloisite 30B and their ability to form strong hydrogen bonds [78]. However, even if the presence of clay induced the degradation of the nanocomposite samples by up to 12% of their original weight, the samples retained their shape during degradation analyses.

Therefore, hydrogel network degradation may be regulated by using different types of nanoclay according to the characteristics of the tissue targeted for regeneration. Additionally, the degradation/erosion reaction can be tuned to obtain desirable release kinetics, enabling the controlled release of polar/unpolar bioactive agents. Nevertheless, it should be noted that degradation products should be non-toxic and adequate for natural clearance [79].

### 3.6. Printability of the Materials

Because the main purpose of this research study was to develop a nanocomposite printing ink that can be used in tissue engineering, four bioinks were investigated to generate biocompatible scaffolds which would allow cells to attach, migrate and proliferate. These inks were based on GelMA hydrogel, which, apart from properties like biocompatibility and biodegradability, benefits from one important property, namely, its structure cell-binding moieties like RGD sequences that promote cell adhesion [33]. However, GelMA ink has the disadvantage of low viscosity, which further causes the collapse of the filaments and the instability of the 3D printed constructs.

In order to improve GelMA properties like viscosity, porosity, cell adhesion and mechanical properties, three types of nanoclays were added to the GelMA matrix, thus new nanocomposite hydrogel–clay printable inks were obtained.

Printability of the hybrid hydrogels was tested using the direct dispensing printhead of the 3D Discovery™ bioprinter from RegenHU Ltd., Villaz-St-Pierre, Switzerland. The direct dispensing printhead uses extrusion technology. Because the extrusion process take place at room temperature, this technology offers the ability to test different materials in a cell-friendly environment.

For the extrusion process, a 3 mL stringe was used with metallic and variable needle dimensions (0.25 mm, 0.33 mm, 0.41 mm, 0.65 mm). Additionally, parameters like pressure (150–600 kPa) and printing speeds (1.2 mm/s to 11 mm/s), were explored in order to establish the most suitable one for each material. BioCAD software was used to design 3D structures, layer by layer. BioCAD is software that allows drawing objects in 2D, and after the G code is generated, the objects can be viewed in 3D. A photocrosslinking step was achieved under UV light, layer by layer, during the printing process.

**Figure 11:**
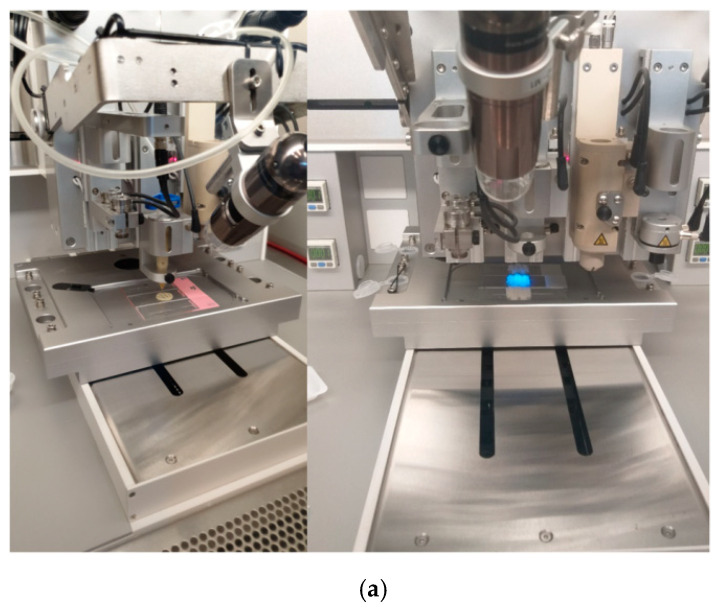

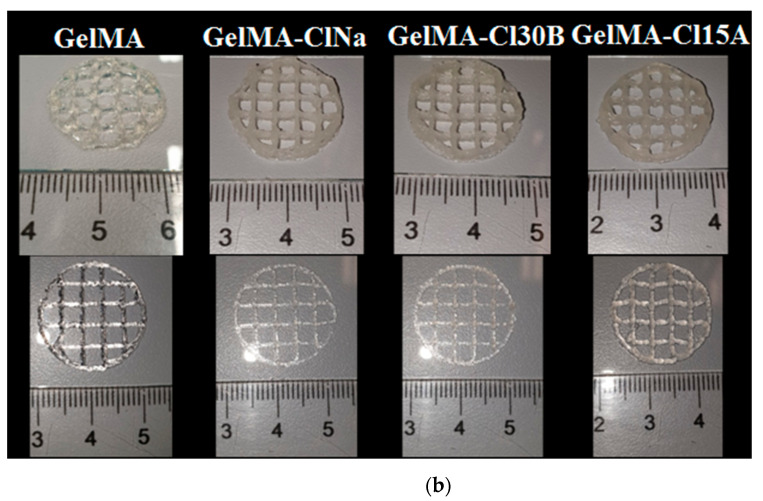
(**a**) 3D printing process. (**b**) The appearance of the 3D printed constructs based on nanocomposite hydrogel inks GelMA, GelMA-ClNa, GelMA-Cl30B, GelMA-Cl15A (5 layers and 1 layer).

Almost all of the printed materials presented regular filaments, and maintained their initial structural integrity (Figure 11).

The low viscosity of GelMA induces instability during the printing process, so final scaffolds showed irregular filaments. In addition, because the material was not very stable, the GelMA filaments collapsed and the final scaffolds did not maintain their initial dimensions.

Because the obtained GelMA-based hydrogel inks presented different viscosities, needles with different diameters were used. After several attempts, a 0.25 mm needle was selected for the printing process of GelMA. As a consequence, scaffolds presented stability and regular forms. Additionally, the final scaffolds maintained their initial printed shape and dimensions.

The current limitations of using GelMA hydrogels in the 3D printing field, such as their process ability and mechanical properties, were modified by using clays. The filaments obtained with GelMA-ClNa ink were regular and the final scaffolds maintained their initial printed shape, too. GelMA-ClNa was printed with a 0.25 mm needle and a pressure of 550 kPa.

Because the organomodified silicates were not uniformly dispersed in the GelMA matrix, during the printing process with GelMA-Cl15A and GelMA-Cl30B inks, the needle clogged occasionally, so the filaments were not continuous as in the other inks. Thus, GelMA-Cl15A and GelMA-Cl30B were printed with a 0.41 mm needle and a pressure of 580 kPa and 300 kPa, respectively.

By using UV irradiation simultaneously with 3D deposition, the accuracy and fidelity of the shape was improved and this also prevented the collapse of subsequent printed layers. The 3D printed structures demonstrated excellent shape fidelity with well-ordered surfaces and continuous thickness. The scaffolds did not present aggregates in the extremities when direction and speed shifted. Moreover, porous and interconnected structures of up to 20 layers in height were printed without collapsing.

Summarizing, the newly developed GelMA inks compounded with different types of clay allowed the printing of 3D constructs with complex geometries which will further serve as a suitable medium for the administration of nutrients and oxygen to target growing tissues.

### 3.7. Scanning Electron Microscopy (SEM) and Micro-CT Analyses

Porosity of the material is one of the main parameters studied in hydrogel-based scaffold, with important consequences on cell viability. The pores facilitate cell encapsulation, proliferation and vascularization and boost nutrient transportation [80].

**Figure 12:**
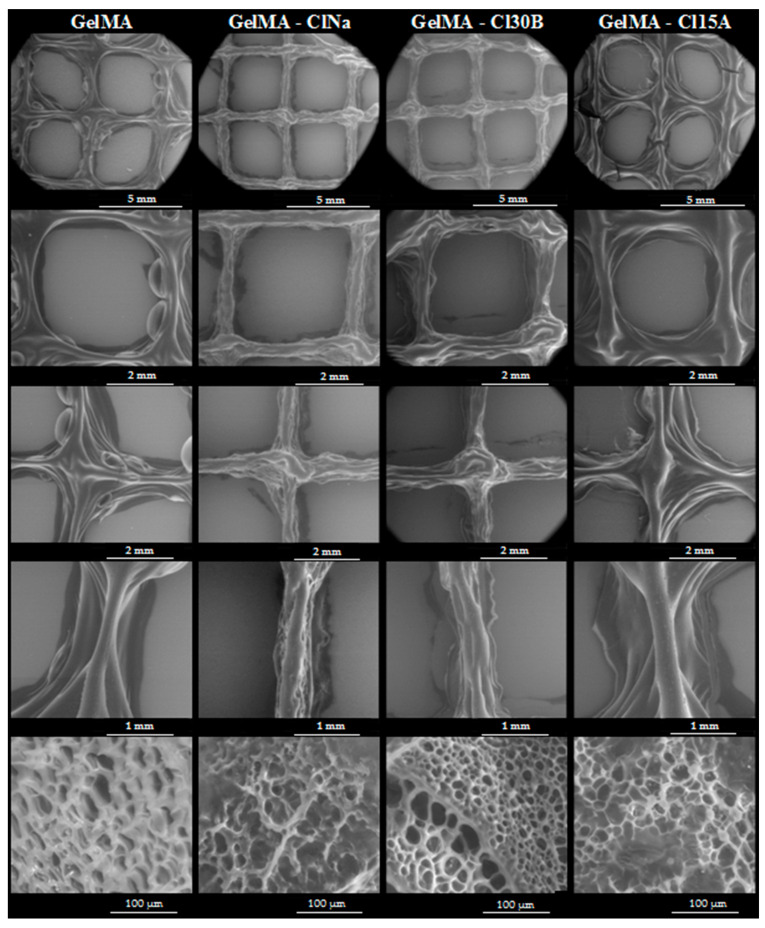
SEM images showing the microstructure aspect of GelMA, GelMA-ClNa, GelMA-30B and GelMA-15A scaffolds (magnitude ×25, ×50, ×50, ×100, ×1000).

All the samples revealed high porosities, as observed from SEM analyses (Figure 12). The GelMA scaffolds present macropores with different sizes separated by smooth thin surface walls. The presence and type of clay nanoparticles obviously affected GelMA morphology. The nanocomposite materials based on GelMA modified with several types of layered silicates also present macropores that are separate by thin surface walls and interconnected macropores. Apparently, clays induce a higher porosity as a consequence of their interaction and distribution within GelMA-based hydrogels.

**Figure 13:**
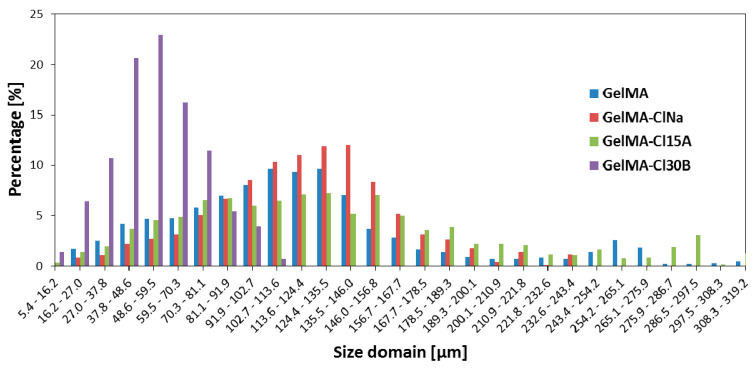
Micro-CT analyses—pore size distribution of GelMA and GelMA-based samples.

The porosity of the material was also confirmed by micro-CT, which showed that the porosity of nanocomposite samples is different depending on the type of clay used in synthesis (Figure 13). Thus, the GelMA-based nanocomposite with ClNa presents the largest and the most uniformly distributed pores. At the opposite end, we have the GelMA-Cl30B sample, which presented the smallest pore dimensions at a high percentage.

**Figure 14:**
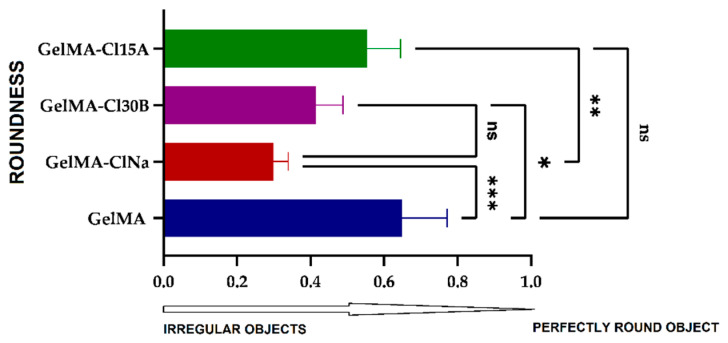
The roundness of open pores calculated for GelMA and GelMA-based 3D printed scaffolds. Statistical significance: ns *p* < 0.5; * *p* < 0.05; ** *p* < 0.005; *** *p* < 0.0005.

The analysis of the roundness of open pores could offer a possible quantification of the printed shapes. Using SEM images and the Wadell equation [81], the average roundness of the open pores of the 3D printed scaffolds was calculated (Figure 14).

For a perfectly round object, a value of R = 1 is obtained, while irregular objects tend to have values less than 1. The highest calculated value of 0.65 was obtained for the GelMA sample when compared with the values obtained for the nanocomposite scaffolds (GelMA-ClNa, 0.29; GelMA-Cl30B, 0.41; and GelMA-Cl15A, 0.55). These noteworthy differences indicate that 3D printed constructs maintained their shape fidelity better in the presence of several types of filler. Layered silicates induced an enhanced structural integrity during printing but also during the UV crosslinking and drying processes. The increase in shape roundness from the GelMA-ClNa sample to GelMA-Cl30B and further to GelMA-Cl15A was closely related to sample viscosities, which showed the same tendency. Therefore, in the case of samples with higher viscosity, the printed form was better preserved.

## 4. Conclusions

Novel hydrogel–nanoclay printing inks and corresponding 3D printed structures have been successfully developed and investigated. The newly developed GelMA inks compounded with different types of clay allowed the printing of 3D structures. The presence of nanoclays induced significant changes in the properties of the biopolymeric inks and, further, in the morphology and structure of the 3D printed nanocomposite scaffolds. Using different types of naturally sourced nanoclays could play a significant role in the final properties of the UV crosslinked GelMA-based 3D structures.

More studies are required to have a better understanding of clay–polymer interactions and how the type and concentration of various natural clays could influence the printing process. Future prospects include the testing of the nanomaterials for biological response but also the encapsulation of bioactive agents in the 3D printed scaffolds.

## Figures and Tables

**Table 1:** The values of the clay characteristic diffraction peak d_001_ at 2θ.

Sample	2θ (°)	d_001_ (Å)
ClNa	6.79	13.02
GelMA-ClNa	4.41	20.00
Cl30B	4.72	18.73
GelMA-Cl30B	4.54	19.45
Cl15A	2.67	32.75
GelMA-Cl15A	2.48	35.60

**Table 2:** Results of Dunn’s test for multiple comparisons.

No.	Comparison	Z	P unadj.	P adj.
1	DataCl15A-dataCl30B	1.07	0.283	0.283
2	DataCl15A-dataClNa	−4.59	4.48 × 10^−6^	6.72 × 10^−6^
3	DataCl30B-dataClNa	−6.76	1.39 × 10^−11^	4.17 × 10^−11^

**Table 3:** Summary of data obtained by measuring the interlamellar distance in BF-TEM images (values are given in Å).

Sample	Min.	1st Qu.	Median	Mean	3rd Qu.	Max.
GelMA-ClNa	15	52.5	79	85.84	111	322
GelMA-Cl30B	16	19	25	27.03	29.5	89
GelMA-Cl15A	13	22	28	31.1	34	66

## Data Availability

Not applicable.
